#  Isolated Laryngeal Amyloidosis

**Published:** 2013

**Authors:** Fatholah Behnoud, Neda Baghbanian

**Affiliations:** 1 *Department of otorhinolaryngology, Hamedan University of Medical Sciences, Hamedan, Iran.*

**Keywords:** Amyloidosis, Hoarseness, Larynx

## Abstract

**Introduction:**

Amyloidosis comprises a heterogeneous group of disorders characterized by the deposition of amyloid protein in various organs of the body. The larynx is one of the rarer sites where amyloidosis occurs.

**Case Report:**

A 36-year-old man presented with a two-year history of hoarseness of voice. He had a positive history of smoking, but no history of long-term consumption of alcohol. Physical examination revealed a pinkish mass about 1.5× 1.5 cm in size on his left False Vocal Cord (FVC) extending to the left arytenoid, which resulted in asymmetry of the posterior larynx. He also had a chronic perforation of the right tympanic membrane with a conductive hearing loss consisting of a 50 dB gap in pure tune audiometry. The FVC mass was excised with a CO_2 _laser and on follow-up his voice got much better, but the hoarseness was not fully resolved.

**Conclusion:**

Amyloidosis of the larynx is a rare, usually benign process but the area is the most common site for isolated amyloid deposits to occur in the head and neck.

## Introduction

Amyloidosis comprises a heterogeneous group of disorders characterized by the deposition of amyloid protein in various target organs of the body. This ultimately leads to organ malfunction and failure (1). Amyloidosis is derived from the Greek words amylon, meaning starch, and eidos, meaning resemblance. Virchow was the first to use the term amyloid because of the starch-like reaction of the protein when treated with iodine and sulphuric acid (2). Amyloidosis commonly affects individuals between 50 and 70 years of age with a male: female predominance of 3:1. However, the youngest case reported so far in the literature was an 11-year-old girl. Amyloidosis of the larynx is a rare, usually benign process but the area is the most common site for isolated amyloid deposits to occur in the head and neck. The deposits account for 0.2 to 1.2% of benign tumors of the larynx (3).

While amyloid is not benign in its systemic form, it usually behaves in a harmless fashion when localized to one site in the head and neck region. Laryngeal involvement has been described at all levels of the larynx. Some authors have stated that the most common location is at the true vocal cord. Other sites include the eye, the orbits, and the major and minor salivary glands, while submucosal deposits have been observed in the nose, paranasal cavities, nasopharynx, oral cavity, stomatopharynx, bronchotracheal tree, and lungs. Oral and paranasal amyloidosis is usually a manifestation of systematic amyloidosis, mainly plasma cell dyscrasia(4).

Patients with amyloidosis of the larynx typically present with hoarseness, although they may present with cough, globus, hemoptysis, stridor, or dyspnea. The deposition of amyloid may occur either diffusely or in a single tumor nodule. Microscopic examination typically shows a diffuse submucosal globular deposition of a largely acellular eosinophilic material that exhibits apple-green birefringence under polarized light when stained with Congo red. There may also be an associated sparse, mixed chronic inflammatory infiltrate consisting of mature plasma cells and lymphocytes (5). Here, we report a case of primary laryngeal amyloidosis.

## Case Report

**Fig 1:**
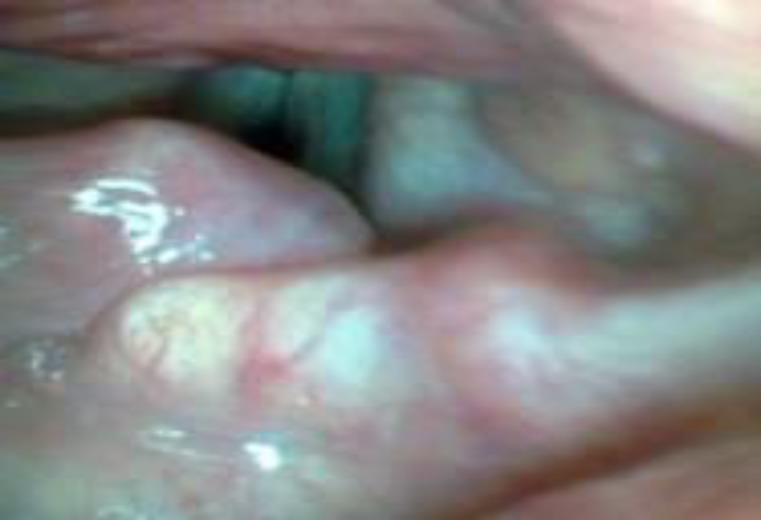
Laryngoscopic view of the amyloid mass

A 36-year-old man presented with a two-year history of hoarseness. He had a positive history of smoking but not of long-term consumption of alcohol. Physical examina- tion revealed a pinkish mass about 1.5 × 1.5 cm in size on his left False Vocal Cord (FVC) extending to the left arytenoid, which resulted in asymmetry of the posterior larynx (Fig 1).

**Fig 2:**
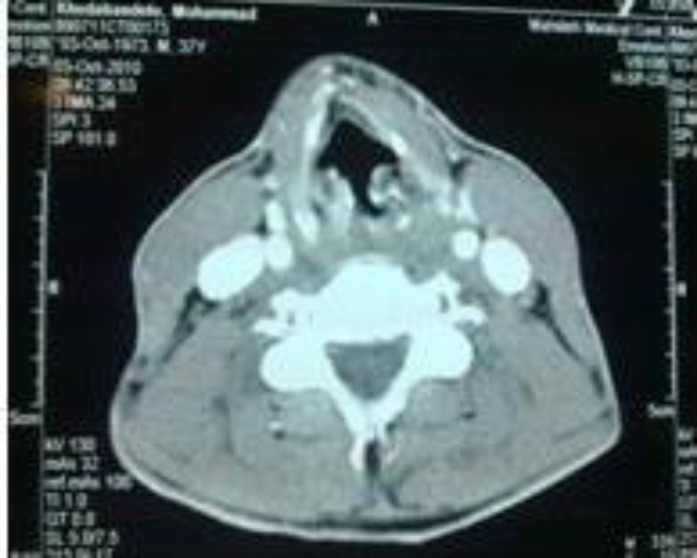
CT scan with contrast but without significant enhancement

He also had a chronic perforation of the right tympanic membrane with a conductive hearing loss consisting of a 50 dB gap in pure tune audiometry. Other systemic examina- tions of the patient were normal, and there was no generalized lymphadenopathy or hepatosplenomegaly. An MRI and CT-scan of the neck were taken in which a mass-like lesion (about 10 mm) without significant contrast enhancement was visible in the left vocal cord and bulging into the airway (Fig 2).

**Fig3:**
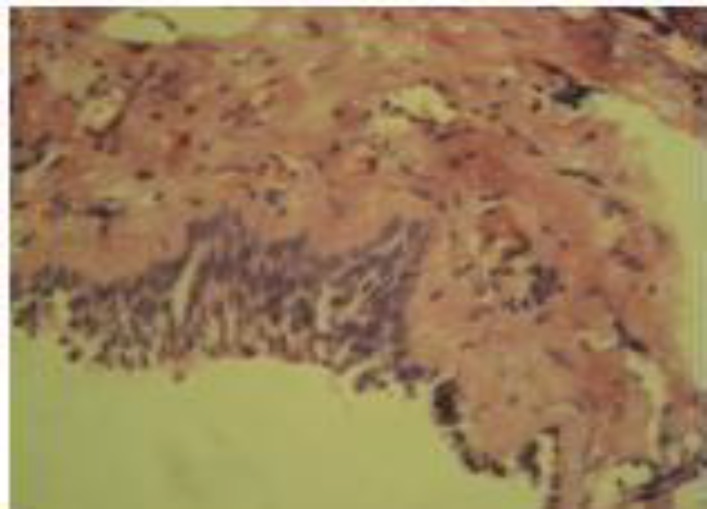
Histopathologic examination showing homogeneous eosinophilic deposits in the stroma

**Fig4:**
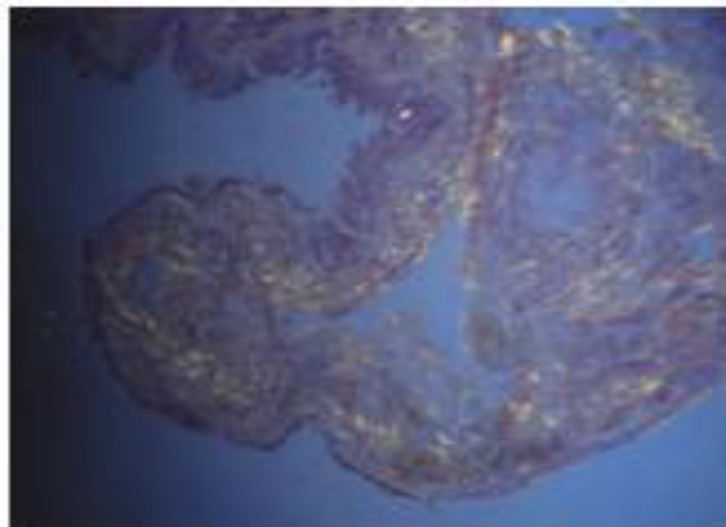
Apple-green birefringence with Congo-red stain confirmed the diagnosis of amyloidosis

The patient underwent direct laryngoscopy and biopsy. The left arytenoid seemed to be dislocated, but attempts to reduce it were unsuccessful. On histopathologic examination homogeneous eosinophilic deposits in the stroma under the epithelium were seen. Apple-green birefringence with Congo-red stain confirmed the diagnosis of amyloidosis. No signs of malignancy or dysplasia were seen (Figs 3,4).

A thorough medical workup including tests such as cell blood count, C-reactive protein, erythrocyte sedimentation rate and purified protein derivatives CBC, CRP, ESR, PPD, liver function tests, rheumatologic tests, urine volume, creatinine and protein, serum BUN and creatinine, bone marrow biopsy and aspiration, and an abdominal wall fatty tissue biopsy was performed. 

The result of serum protein electrophoresis was normal and in electrocardiography and echocardiography, cardiac involvement was ruled out. Sonography of the abdomen and pelvis was also normal and the workup revealed no evidence of amyloid outside the larynx. Finally, the FVC mass was excised with a CO_2 _laser and on follow-up the patient’s voice got much better, but the hoarseness was not fully resolved.

## Discussion

Two theories have been proposed to explain localized amyloidosis of the larynx. The first suggests the occurrence of a plasma cell reaction to inflammatory antigens and is supported by pathologic studies showing the presence of mixed polyclonal plasma cells interspersed with the amyloid tissue. Another, more likely scenario, points to the inability of the body to clear light chains produced by plasma cells located in the mucosal-associated lymphoid tissue (6). 

MRI is the technique of choice to detect the most specific features of amyloidosis, since amyloid deposits present an intermediate T1-weighted signal intensity and low T2-weighted signal intensity (7).

When a diagnosis of laryngeal amyloidosis is made, workup should include studies to rule out systemic disease, as well as an accurate assessment of the laryngeal involvement. Multiple myeloma, rheumatic diseases, and tuberculosis are some of the systemic causes that must be considered. Amyloidosis associated with familial syndromes and endocrinopathies, such as medullary thyroid cancer, also needs to be investigated. 

The workup should include a pulmonary evaluation; tuberculin skin test; complete blood cell count; blood urea nitrogen and creatinine levels; liver enzyme studies; sedimentation rate; determination of Rh factor; urinalysis; antinuclear antibody values; and serum and urine immune- electrophoresis. For most cases of suspected localized amyloidosis it is probably not necessary to investigate for systemic disease with biopsies of the lip, rectum, or abdominal fat, because of the low yield. However, fine-needle aspiration of abdominal fat has been shown to be a simple and effective procedure to rule out systemic amyloidosis (3). 

Our patient had a pinkish mass about 1.5 × 1.5 cm in size on the left FVC extending to the left arytenoid, which resulted in asymmetry of posterior larynx (Fig. 1). An MRI and CT-scan of the neck were taken in which a mass-like lesion (about 10 mm) without significant contrast enhancement was identified in the left vocal cord and bulging into the airway (Fig 2). The FVC mass was excised with CO_2 _laser and on follow-up his voice got much better, but the hoarseness was not fully resolved.

Treatment of laryngeal amyloidosis varies from simple observation of the lesion to partial laryngectomy according to the extension of the disease into the larynx. Endoscopic CO_2_ laser excision of the mass should be the first choice of therapy. The course of the condition under discussion is slow but sudden relapse is possible (7).

Localized lesions can be completely removed endoscopically either via cold knife or CO_2 _laser (8). Surgical resection using a CO_2_ laser is very effective for removal of diseased tissue. Other treatments options that have been described include corticosteroids, radiotherapy, and agents like colchicine and melphalan. However, these modalities have yielded variable results (9,10).

In a study on 16 patients with amyloidosis, Pribitkin and colleagues reported the amyloidosis of the upper aerodigestive tract generally behaves as a benign, localized condition treatable by surgical resection. Regular follow-up with laryngoscopy is indicated for early diagnosis of recurrence, and multiple surgical procedures may be required to control symptoms (11). However, Herbert and Krzysztof studied 10 patients with isolated laryngeal amyloidosis. Their study revealed amyloidosis causes a smooth submucosal swelling of the false vocal fold and true vocal cord and that no effective medical treatment will remove the deposits (12).

## Conclusion

Amyloidosis of the larynx is a rare, usually benign process but the area is the most common site for isolated amyloid deposits to occur in the head and neck. When a diagnosis of laryngeal amyloidosis is made, workup should include extensive studies to rule out systemic disease, as well as an accurate assessment of laryngeal involvement.
